# On the Electronic Structure of 2H-MoS_2_: Correlating DFT Calculations and In-Situ Mechanical Bending on TEM

**DOI:** 10.3390/ma15196732

**Published:** 2022-09-28

**Authors:** Manuel Ramos, Oscar A. López-Galán, Javier Polanco, Miguel José-Yacamán

**Affiliations:** 1 Departamento de Física y Matemáticas, Instituto de Ingeniería y Tecnología, Universidad Autónoma de Ciudad Juárez, Edificio G-301A, 450 Avenida del Charro, Ciudad Juárez 32310, Chihuahua, Mexico; 2 Applied Physics and Materials Science Department and Center for Material Interfaces Research and Applications (MIRA), Northern Arizona University, Flagstaff, AZ 86011, USA

**Keywords:** MoS_2_, band structure, isosurface, DFT, HOMO, LUMO

## Abstract

We present a systematic density functional theory study to determine the electronic structure of bending 2H-MoS_2_ layers up to 75° using information from in-situ nanoindentation TEM observations. The results from HOMO/LUMO and density of states plots indicate a metallic transition from the typical semiconducting phase, near Fermi energy level (*E_F_*) as a function of bending, which can mainly occur due to bending curvatures inducing a stretching and contracting of sulfur-sulfur chemical bonds located mostly over basal (001)-plane; furthermore, molybdenum ions play a major role in such transitions due to reallocation of their metallic *d*-character orbitals and the creation of “*free electrons*”, possibly having an overlap between Mo_-dx_^2^_-y_^2^ and Mo_dz_^2^ orbitals. This research on the metallic transition of 2H-MoS_2_ allows us to understand the high catalytic activity for MoS_2_ nanostructures as extensively reported in the literature.

## 1. Introduction

Molybdenum disulfide (MoS_2_), which is a mineral known as molybdenite, has attracted much attention in the past two decades in the materials community due to its extensive usage ranging from use as a high vacuum lubricant [1], hydrodesulfurization catalyst [2,3], and two-dimensional channel material for field-effect transistors [4,5]. The sandwich-like chemical structure was resolved by chemist Linus Pauling and because of its two-dimensional characteristics, is considered analog to graphene with an indirect or direct band gap between 1.67 eV and 1.8 eV for bulk and single layers, respectively [6,7]. In bulk, layers are stacked along the c-axis with an interlayer separation of 6.2 Å and held together by weak Van der Waals forces; its semiconductor character is caused by chemical bonding between two chalcogen sulfurs to one molybdenum disposed in an octahedral geometry [8]. However, even when layers are planar sheets, several reports indicate MoS_2_ can atomically pack in diverse shapes and morphologies (i.e., nanotubes, nanoclusters, and nanospheres) and have different electrical behaviors, which is mainly influenced by the chemical synthesis method and the resulting morphology [9,10]. As reported in the literature, using density functional theory methods, it is possible to achieve a deep understanding of the particular aspects of the electronic structure in 2H-MoS_2_, ranging from catalytic to transport properties [11,12,13]. Ghorbani-Asl et al. calculated energy band gaps of MoS_2_, WS_2_, and NbS_2_ when tensile-strain is present, revealing a semiconductor to metallic transition for layer bending by 11% [14]. Sharma et al. determined intrinsic change in the energy gap of transition metal dichalcogenides at 2–4% of externally applied strain with maximum stress load in a range between 9 GPa to 12 GPa [15]. In the case of MoS_2_, the energy band gap variation from 1.8 eV to 0.8 eV can occur due to the intercalation of MoS_2_ layers with tungsten diselenide (WSe_2_) [16]. MoS_2_ can also present Moiré patterns, which are formed due to layers rotated at 12° with intrinsic semiconductor-to-metal transition, being determined by density functional calculations as well as by high-resolution transmission electron microscopy (HRTEM) observations [17]. Beyond conventional electron microscopy, nowadays in-situ techniques have attracted much attention, especially for the evaluation during observations of fundamental aspects such as mechanical strength using TEM sample holders adapted with AFM nanoindenters [18,19]. Previous nano-indenter measurements indicate that 2H-MoS_2_ thin films have an average hardness of 10.5 ± 0.1 GPa and elastic modulus of 136 ± 2 GPa [20], similar to studies completed by Lahouij et al. on onion-like MoS_2_ nanoparticles, which demonstrate strong evidence of tribological properties for normal forces of 100 nN and 400 nN with an experimental values contact pressure of 20 MPa to 90 MPa [21]. Here, we present a systematic study on the bending behavior of a 2H-MoS_2_ slab in the <001> direction employing density functional calculations. We aim to determine the electronic structure in 2H-MoS_2_ and how it is influenced by mechanical bending. With this, we achieve a theoretical understanding of localized “*free-electrons*” in terms of curved nanostructures and the characteristics of mechanical resilience as observed by in-situ TEM [22]. We present information on the highest occupied molecular orbital and the lowest unoccupied molecular orbital (HOMO and LUMO) energy levels localization when the 2H-MoS_2_ layer is bent, as well as its electronic structure by the density of states near energy Fermi level (*E_F_*). Finally, we correlated the electrical properties of the bent 2H-MoS_2_ slab as a viable material for catalytic and energy storage based on our results.

## 2. Methods

### 2.1. Computational Methods

**Figure 1:**
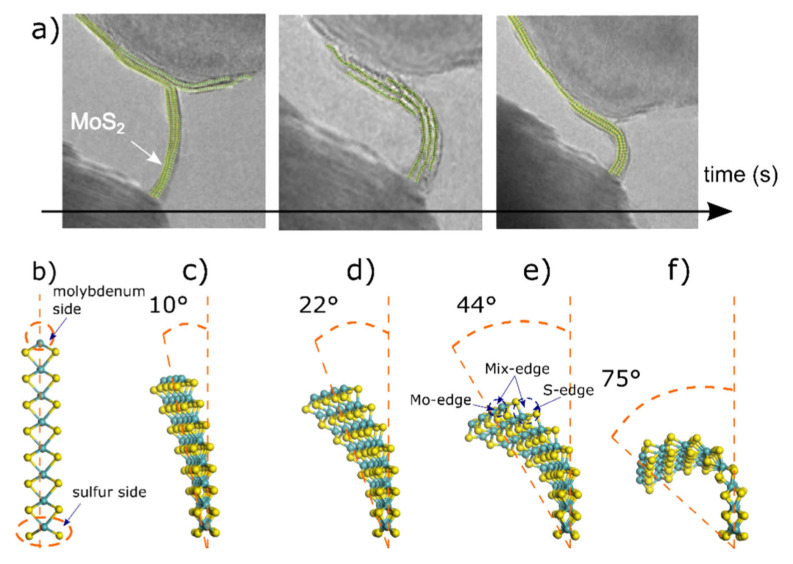
(**a**) Color-coded experimental observations made by in-situ HRTEM technique showing the induced bending curvature for 2H-MoS_2_ layers, adapted from Casillas et al., J. Phys. Chem. C 2015, 119, 710−715. Ref. [22] under rights and permissions of ACS copyrights©. (**b**–**f**) Molecular models for 2H-MoS_2_ are used for HOMO-LUMO calculations; the main bending angles from 0° to 75°. Cyan balls are molybdenum atoms and yellow balls are sulfur atoms. Mo side refers to the upper part of the slab composed of fourfold molybdenum ions. S-edge refers to the bottom part of the slab which ends in sulfur ions with twofold coordination.

Our computational study was completed using a conventional 2H-MoS_2_ molecular layer containing 117 atoms and having dimensions of 22 Å × 10 Å in length and width; no periodic conditions are considered. This layer was cut in the 001 direction from a unit cell of a hexagonal 2H-MoS_2_ (space group P6_3_/mmc) with lattice parameters of a = b = 3.1 Å, and c = 18.4 Å. Then, by using the open access Avogadro Chemistry^®^ package [23], a series of molecular dynamics simulations comprising the 2H-MoS_2_ model was performed, from which frames of the molecular model while bending were exported to the Materials Studio© package, focusing only on 10°, 22°, 44°, and 75° bending angles. These bending angles match observations made on in-situ TEM, as schematically described in Figure 1.

HOMO and LUMO energy levels localization was calculated by density functional theory (DFT) code Dmol^3^ implementing the Generalized Gradient Approximation (GGA) and Perdew–Burke–Herzenhof (PBE) as exchange-correlation functional; for single point energy calculations a self-consistent field (SCF) convergence criteria of 1 × 10^−6^ eV/atom under double numerical polarization (DNP) basis set was used; all species were treated with effective core potentials and unrestricted spin with thermal smearing value of 0.027 eV (0.001 Ha) together with a long-range dispersion correction DFT-D2 [24].

### 2.2. Experimental TEM

Transmission electron microscopy was performed using a JEOL 2010 (Akishima, Japan) equipped with a field emission electron beam at 200 kV. Detailed descriptions of MoS_2_ preparation can be found in previous works by the author [3]. AFM-TEM mounting and the proper in-situ experiment were performed following the methodology described by Casillas et al., where micrographs were recorded in a bottom-mounted AMT CCD with an exposure time of 0.1 s [3,22].

## 3. Results and Discussion

### 3.1. HOMO and LUMO at Bending Angles

We verify that Mo-S bond length is reduced by 25% at the bending angle of 75°and that curving effects between energy levels occur in intensity by comparing the resulting electronic structure. As a result, we can compare the resulting geometry of the layer by estimating the cord length, radius, and arc length of the layer (Table 1). The resulting values are in the same order of magnitude as those encountered experimentally, confirming the validity of the methodology used for the mechanical bending of the 2H-MoS_2_ molecular layer. The mean Mo-S bond length is reduced from 2.4 Å to 1.2 Å when the slabs go from 0° to 75°, which may result in strong interaction between s, p, and d orbitals, causing direct influence on the electronic structure of 2H-MoS_2_, as suggested previously by Bhattacharyya et al. [24]; this bond length contraction is also translated in a contraction on the dimension of the layer in the vertical direction, parallel to the plane 001, which is equivalent to the computed cord length.

**Figure 2:**
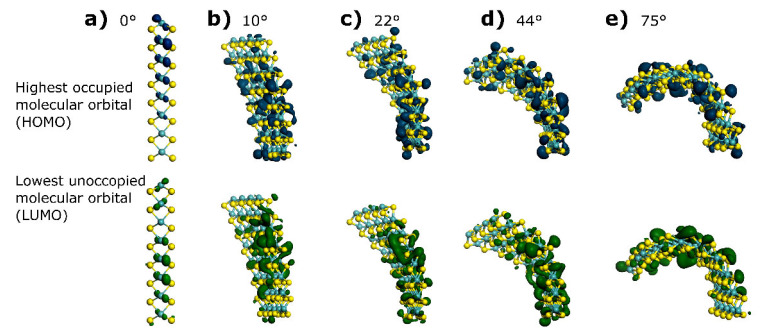
Energy isosurfaces for HOMO (dark blue isosurface) and LUMO (dark green isosurface) computed for theoretical models as a function of slab bending, isosurface value is 0.03. Cyan balls represent the position of molybdenum and yellow balls represent sulfur. It is possible to determine that bending effects provoke changes in the 2H-MoS_2_ conventional electronic structure, which has been highly discussed in the literature, (**a**) Represents the HOMO-LUMO distribution for 0° angle 2H-MoS_2_ layer, one can observed not much delocalized electrons clouds. (**b**–**e**) The 2H-MoS_2_ layers are bend from 10° to 75° and it was possible to detect a perturbation of HOMO-LUMO energy states by delocalized electron clouds.

In the HOMO-LUMO plots, energy levels localize as density profiles as presented in Figure 2. The dark blue isosurface presents HOMO distribution, which is analogous to the valence band energy level, whereas the dark green isosurface corresponds to LUMO distribution and is analogous to the energy level known as the conduction band. We observe that differences arise between energy levels as a function of slab bending: HOMO contribution comes mainly from molybdenum for bending angles of 10° and 20° and, at 44°, electron orbitals disperse, and high electron clouds are concentrated at the middle and bottom sections of the structure. When reaching a 75° bending angle, HOMO isosurfaces seem to concentrate mainly in the middle and top section of the structure; electron density isosurfaces are mixed with contributions coming from sulfur sites on the structure, which suggests the electronic contribution from molybdenum to the HOMO varies from 60% to 40% when molybdenum and sulfur get closer due to bond length reduction, which is in agreement with observations made by Ding et al., who calculated energy isosurfaces for edge S-Mo corner sites in 2H-MoS_2_ [25].

Now, when inspecting LUMO energy density plots, it is possible to detect that the distribution of the orbital remains mainly at the center site of the 2H-MoS_2_ layer at angles of 10° and 22°, and also mixed molybdenum and sulfur contribution was encountered. For bending angle values of 10°, the LUMO distributes around molybdenum ions all over the layer, providing theoretical proof that the majority MoS_2_ conduction band consists of molybdenum *d*-character orbitals, as described by Yazyev & Kis [26]. However, LUMO distribution appears to have a more even distribution between molybdenum and sulfur sites, occurring at 22° and 44° bending angles, and at 75°, LUMO density isosurfaces concentrate at the upper part of the layer, where bonding stretch is more severe, allowing us to understand this chemical-physical effect as a mixed contribution from molybdenum and sulfur to the electronic structure, similarly to the HOMO isosurface. When inspecting closely edge sites, it is possible to see that HOMO distribution increases as bending occurs on both the molybdenum and sulfur sides, and more strongly at 75°. On the other hand, LUMO variation implies large electron migration from the bottom to the upper part of the layer at 44° to 75°, causing a high concentration of LUMO at the molybdenum side for d-character orbitals. When S-S bonds contract, they cause an increase in LUMO as displayed in Figure 2, causing higher electron density at the K point, where clearly the density of states indicates the valence band cross-energy Fermi (*E_F_*) level [14].

**Figure 3:**
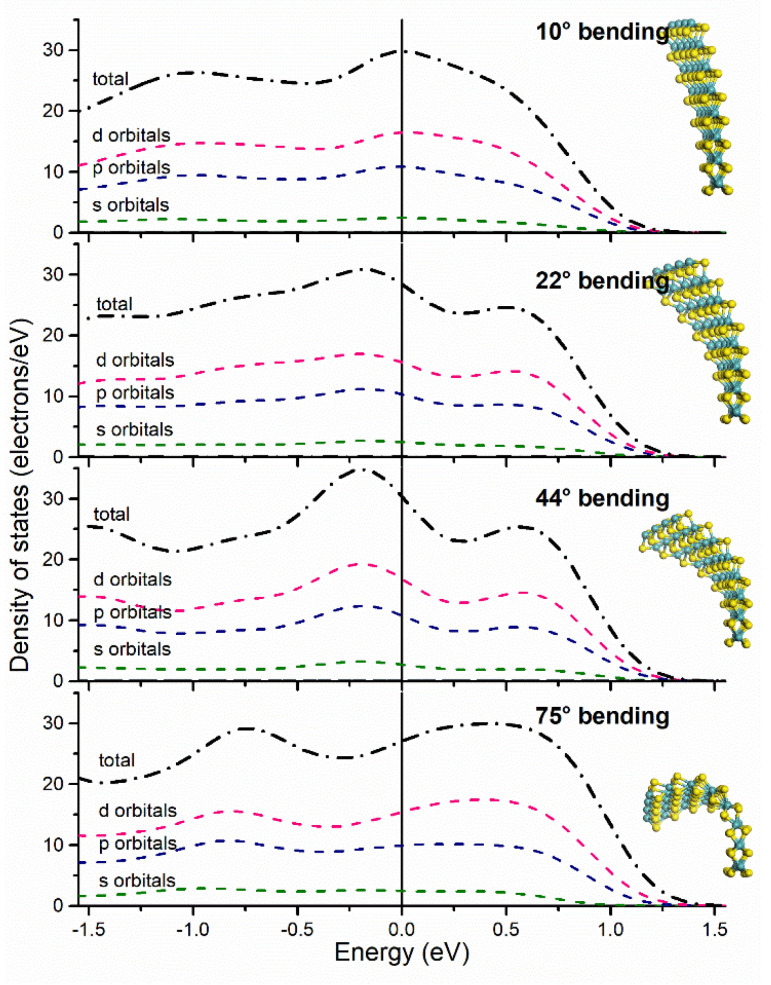
Computed partial and total density of states for all bending curvatures angles plotted near the Fermi energy level set at 0 eV. Data indicate the electronic structure crosses *E_F_* which is characteristic of semi-metallic and metallic electronic structures, the transition highly discussed by others [17,26,27].

HOMO and LUMO energy positions have a more negative value at a bending angle of 44° compared to their energy positions at a bending angle of 75° (Table 2). Calculating the difference between HOMO and LUMO energy position as ΔE, we found that at 44°, we have a maximum value, while it decreases until a minimum at a 75° bending angle. In addition, the integration of the total density of states from −0.5 eV to 0.5 eV (Figure 3) results in a theoretical availability of electrons near E_F_, which is higher at 0° and at 44° compared to the other bending angles. This is explained by recalling the previous results on ΔE, which may indicate that if the two levels are more separated, electron wave functions are less prone to overlap and thus, populate more states near *E_F_*. At 0° of bending (unstrained molecular layer) we have an estimation of 30 electrons in the vicinity of *E_F_* (Figure S1), which is the highest of all situations. However, this higher availability of electrons does not translate into a direct improvement in the catalytic activity as will be discussed in the next section. The high electron availability at 44° suggests an enhanced electron flow if an applied voltage is set, which could be used as a bend-modulated contact in flexible electronics [27].

### 3.2. Electronic Structure by Partial Density of States

The calculations for different bending angle curvatures in 2H-MoS_2_ to obtain both partial and total density of states (PDOS, DOS) reflect metallic or semi-metallic behavior for angles as low as 10° of bending curvature (Figure 3). In the proposed 2H-MoS_2_ computational model, metal ions have an octahedral bonding geometry while sulfur ions have a sp^3^ hybridization type, observed for all DOS energy plots for s and p orbitals distribution. It is worth noting that the sp^3^ hybridization is more pronounced at 44° of bending curvature. Notably, *d*-character orbitals from molybdenum present a similar energy DOS profile when compared with p-character orbitals, indicating that d and p orbitals are interacting inside the layered structure due to stretching/contracting of its chemical bonds, as observed by in-situ TEM experiments as described by Casillas et al. [22]. The orbital distribution behaves as a continuum throughout the vicinity of E_F_ -as well as further away as depicted in Figure S2, indicating a semi-metallic character when the layer is high under bending effects at a mechanical bending when compared to a pristine (unbent) layer with out-of-plane stress [28]; previous studies found a similar behavior but on the compressive strain of a MoS_2_ unit cell by 14% [27].

**Figure 4:**
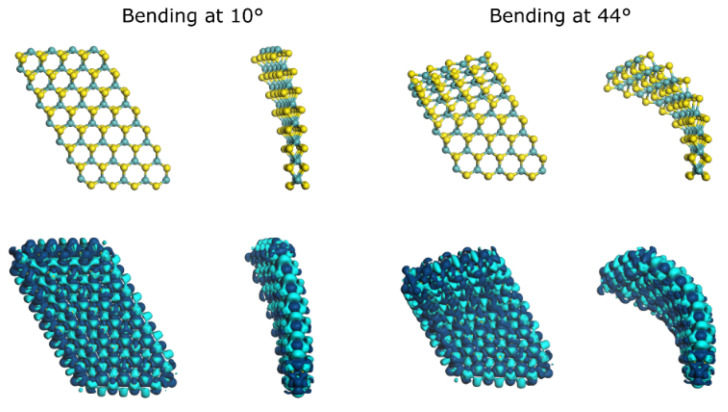
Projection of electron density surface for both bending curvatures angles 10° and 44°. The light blue surface represents positively charged density and navy blue represents negatively charged density. One can observe electron density is more packed as the bending curvature angle increases and the negatively charged density increases at Mo-edge.

Moreover, when the layer undergoes bending curvature from 0° to 44°, Mo_-dx_^2^_-y_^2^ and Mo_dz_^2^ orbitals overlap, with the possibility of maximizing the localized electron cloud. Below and above the bending curvature angle of 44°, orbital overlap causes electron interference, degrading electron localization near the Fermi energy level. Thus, when comparing electron density projection at bending curvature angles of 10° and 44°, electron migration is observed as presented in Figure 4, which graphically indicates that the curvature molecular structure could be more reactive for chemical processes, including hydrodesulfurization as extensively described by Nogueira et al. [29]. However, our study is not deeply focused on theoretical mechanical aspects such as strain–stress relationships as discussed in relation to the 2H-MoS_2_ model elsewhere [30]. At the unstrained condition of the molecular layer and due to the relatively large difference between the HOMO and LUMO levels, an optimal maximization of overlapping of orbitals cannot occur, attributed to the higher population of electrons around E_F_; this has a direct consequence on the catalytic character of the 2H-MoS_2_ molecular layer. Our study allows us to understand and achieve a theoretical understanding of localized “free-electrons” in terms of bond length on curved nanostructures and the characteristics of mechanical resilience as observed by in-situ TEM, perhaps with its advantage for sensors or actuators for applications in the field of nano-electronics.

### 3.3. Catalytic Reactivity in 2H-MoS_2_ Curved Layers

Furthermore, to provide more information about metallic transition when the 2H-MoS_2_ is curved, we proceed to achieve DFT calculations related to relative energy differences due to adsorption of hydrogen on molecular model bending angles of 0°, 10°, and 44° to compare quantitatively reactivity for three main scenarios. Three adsorption sites named S-edge, Mo-S-edge, and Mix-edge were selected. The computed hydrogen adsorption energy (E_ads_) was found using Equation (1).

E_ads_ = E_(slab+hydrogen)_ − E_slab_ − E_hydrogen_ (kcal mol^−1^) (1)

With E_slab + hydrogen_ corresponding to the energy of slab with adsorbed (and dissociated) hydrogen molecules, E_slab_ is the total energy of slab before reaction, and E_hydrogen_ is the energy of diatomic hydrogen. Hence, a negative value of E_ads_ indicates an endothermic process, while a positive value indicates an endothermic reaction.

**Figure 5:**
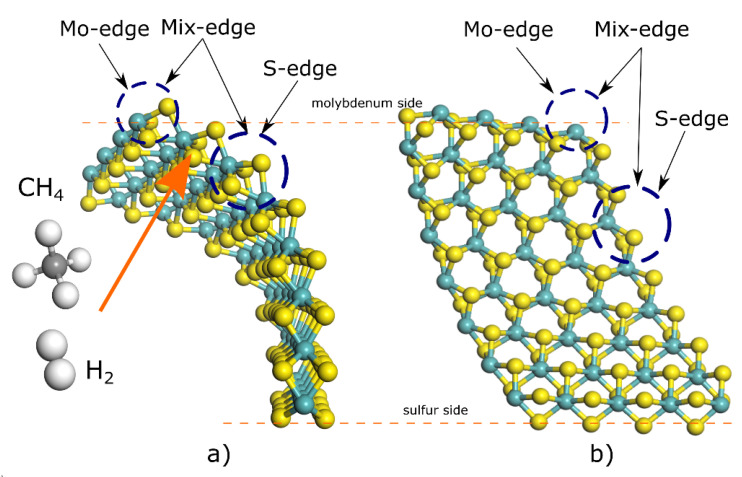
Schematic diagram for 2H-MoS_2_ layer bent at 44° and selected sites for hydrogenation and carburization using H_2_ and CH_4_ as hydrogen and carbon sources. Color code: White is hydrogen, and gray is carbon. (**a**) The side view (101) of 2H-MoS_2_ layer bend at certain angle to indicate the Mo and S edge sites of adsorption. (**b**) The top view (002) of 2H-MoS_2_ layer bend at certain angle to indicate the Mo and S edge sites of adsorption.

The results show bending influences the reactivity of 2H-MoS_2_ towards hydrogen adsorption, showing that in all sites a reduction in adsorption energy is about 50% for a layer bent at 44° compared to adsorption for a layer bent at 10°, and a dramatic decrease in energy adsorption compared to the unstrained molecular layer (Table 3). The results here indicate that the most favorable place for hydrogen adsorption is the S-edge site, requiring 22.95 kcal mol^−1^ for a 2H-MoS_2_ bent at 44° (schematically described in Figure 5). On the contrary, adsorption energies in the unstrained situation have poor performance, requiring almost five times more energy to carry out the same process. The latter is highly correlated to orbital redistribution by bending, which causes dense localized electrons mainly from p-levels, allowing the creation of sp hybridization and π bonds between sulfur from the 2H-MoS_2_ layer and hydrogen molecule.

For sulfur substitution with carbon atoms, which is considered a carburization process as discussed by others [31,32], the substitution energy for CH_4_ species when reacting with the 2H-MoS_2_ layer was estimated using Equation (2); the reaction considers that H_2_ and H_2_S are released as byproducts after sulfur substitution with carbon atoms.

E_ads_ = E_(slab+C)_ + nE_(H__2S)_ + (1/2ny − n)E_(H__2)_ − E_slab_ − nE_(CHy)_ (kcal mol^−1^) (2)

In the above equation, E_(slab+C)_ is the energy of a 2H-MoS_2_ curved layer with n carbon atoms substituting n-sulfur atoms, E_(H__2S)_ is the energy of H_2_S, E_(H__2)_ is the energy of diatomic hydrogen, and E_slab_ is the total energy of layer before the reaction. E_(CHy)_ is the energy of the carbon source, and y corresponds to the number of hydrogen atoms in the carbon source. Here again, a negative value of E_ads_ indicates an endothermic process, and thus, a spontaneous one, while a positive value indicates an endothermic process. Results indicate that the energy demand for sulfur substitution by carbon is higher for 2H-MoS_2_ when it is bent at 44° compared to when the layer is curved at 44°, which is in contrast to the previous results on hydrogen adsorption (Table 3). In the unstrained situation at 0° of bending, carbon substitution is highly endothermic, in agreement with previous calculations on bent Co_9_S_8_/MoS_2_ interfaces [31]. Sulfur removal and carbon substitution cause a change of 3p^4^ to 2p^2^ orbitals, meaning it is translated to the formation of weaker π and σ bonds, which is strongly related to previous experimental reports that indicate onion-like MoS_2_ particles possess important weakening of molybdenum–sulfur chemical bond strength [29,31,32]. In addition, these weakened bonds, which result from a higher disposition of free electrons under bending conditions, could help in the formation of defects, such as sulfur vacancies, which have been observed to promote the catalytic character of MoS_2_ [33].

## 4. Conclusions

Based on information from an in-situ HRTEM study related to mechanical deformation of 2H-MoS_2_ layers as reported by Casillas et al. [22], we are presenting a density functional theory study that described electronic structures corresponding to 2H-MoS_2_ layers when they are curved, which correlates HRTEM observations. Using Dmol^3^© code the calculated HOMO and LUMO and partial density of states indicate that a layer with a bending angle of 44° possesses a large amount of localized “*free electrons*” near the Fermi energy level. Moreover, a metallic transition occurs when mechanical deformation is present, which is caused due to stretching and contracting sulfur to sulfur bonds localized in the (001)-basal plane. This transition is easily translated into an increase in chemical reactivity mainly for hydrogen adsorption and carbon substitution; at sulfur sites, 50% less energy is required for bending angles of 44° as compared to bending angles of 10°. Lastly, the resilience character of 2H-MoS_2_ as described in the in-situ HRTEM experiment was also found in the molecular dynamics simulations using the Avogadro© 1.2.0 package displayed in the video provided in Supplementary Material Video S1.

## Figures and Tables

**Table 1:** Obtained cord length, radius, and arc length for proposed bending curvatures.

Bending Angle	Bending Angle (Radians)	Cord Length (Å)	Radius (Å)	Arc Length (Å)
**10°**	0.174532925	12.25	70.27	12.26
**22°**	0.383972435	11.97	31.36	12.04
**44°**	0.767944871	11.32	15.10	11.60
**75°**	1.308996939	10.27	8.43	11.04

**Table 2:** The HOMO and LUMO computed values (units eV) for bending angles. ΔE is the energy difference between HOMO and LUMO levels. DOS integration was computed from −0.5 eV to 0.5 eV.

Bending Angle	HOMO (eV)	LUMO (eV)	ΔE (eV)	DOS Integration (e^−^)	Fermi Level Crossing Point (e^−^/eV)
0°	−4.833	−4.633	0.2	30	32
10°	−3.67173	−3.61125	0.060480	26.8	29
22°	−3.77025	−3.70124	0.069012	27.1	28
44°	−3.85106	−3.77579	0.075276	28.7	30
75°	−3.69625	−3.64605	0.050193	27.2	27

**Table 3:** Comparison of computed adsorption energy of hydrogen (H_2_) on S-edge, S-Mo-edge, and Mix-edge sites between 2H-MoS_2_ slabs with 10° and 44° and 0° bending.

	Absorption Energy on 44° (E_ads_ /kcal mol^−1^)	Absorption Energy on 10 ° (E_ads_ /kcal mol^−1^)	Absorption Energy on 0 ° (E_ads_ /kcal mol^−1^)
H_2_ (@S-edge)	22.95	43.75	No convergence found
H_2_ (@Mo-edge)	100.00	185.34	847.5
H_2_ (@Mix-edge)	93.91	169.07	5085.9
C (@S-edge)	−1.82 × 10^3^	−1.99 × 10^3^	5.17 × 10^3^
C (@S-edge) (2)	−1.97 × 10^3^	−2.22 × 10^4^	5.18 × 10^3^

## Data Availability

Not applicable.

## Supplementary Materials

The following supporting information can be downloaded at: https://www.mdpi.com/article/10.3390/ma15196732/s1, Figure S1: Total density of states of 2H-MoS_2_ molecular layer with no bending. Figure S2: Total and partial density of states as a function of mechanical bending. Video S1: Resilience 2H-MoS_2_ by direct HRTEM and Molecular Dynamics. Supplementary Material Video S1 corresponds to a digital video taken during the in-situ mechanical deformation using a nano-indenter on the sample holder. The video presents the deformation of 2H-MoS_2_ layers prepared by the exfoliation method as described by Casillas et al. [22]. The molecular dynamics simulation was performed using the open-source computational package Avogadro© 1.2.0 [23] using an 2H-MoS_2_ molecular model.
