# Supplementary material for: On the Electronic Structure of 2H-MoS2: Correlating DFT Calculations and In-Situ Mechanical Bending on TEM
Source: Materials (Basel). 2022 Sep 28;15(19):6732. doi: 10.3390/ma15196732 (PMC9571706; doi:10.3390/ma15196732)
Supplement: Supplementary file 1 [file materials-15-06732-s001.zip › materials-1856631-supplementary.pdf]

On the electronic structure of 2H-MoS_2: Correlating observations made by in-situ mechanical bending on TEM

Manuel Ramos, Oscar A. López-Galán, Javier Polanco, Miguel José Yacamán

SUPPLEMENTAL MATERIALS

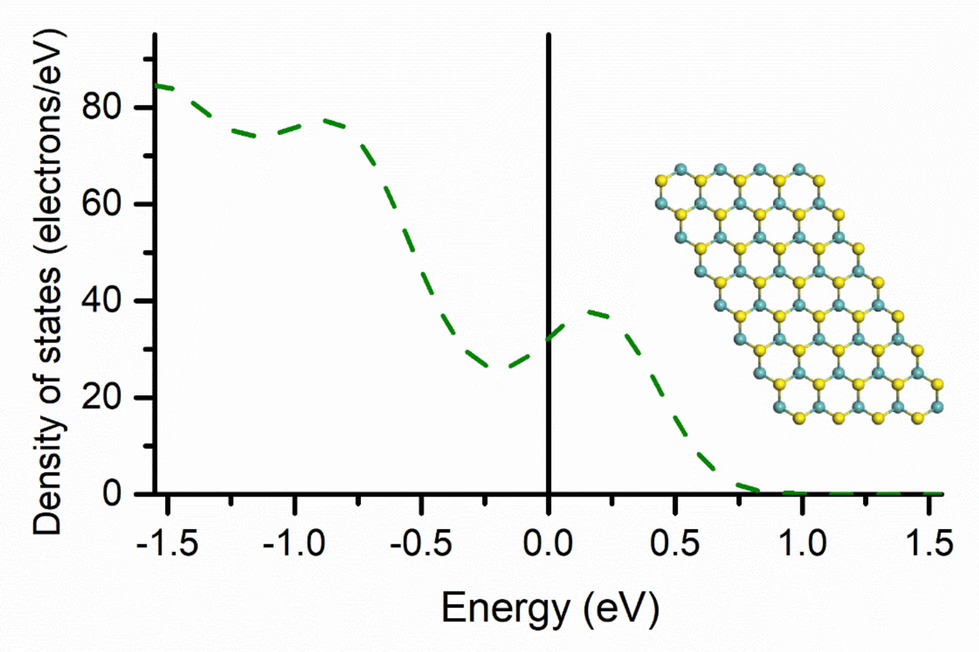

Figure S1. Total density of states of the 2H-MoS_2 molecular layer with no bending. Cyan balls represent the position of molybdenum and yellow balls represent sulfur.

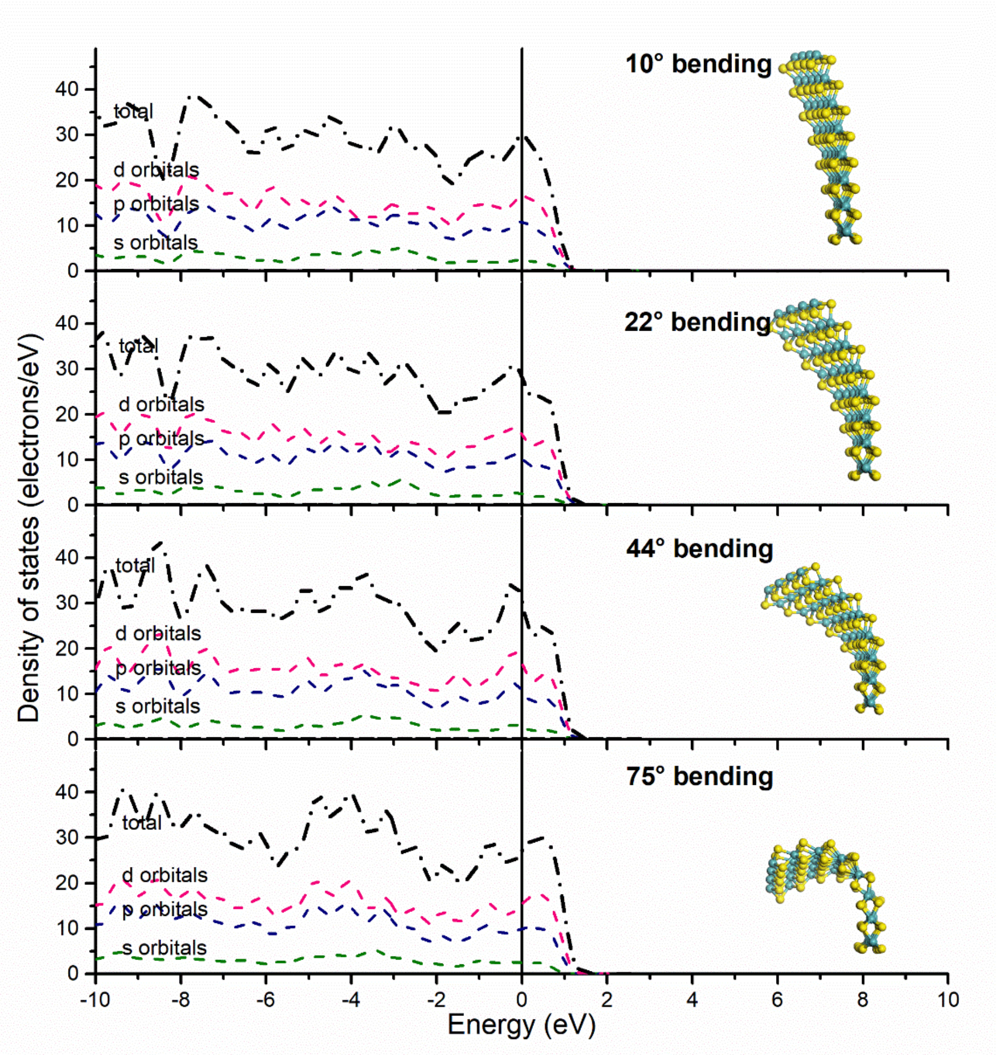

Figure S2. Total and partial density of states computed for the MoS_2 molecular model at 10°, 22°, 44° and 75° of mechanical bending from -10 eV to 10 eV. The observations made by in-situ HRTEM technique, showing the induced bending curvature for 2H-MoS_2 layers, were used to extract the molecular models used for the calculations. Cyan balls represent the position of molybdenum and yellow balls represent sulfur.
